# Exosomal Proteins and Lipids as Potential Biomarkers for Lung Cancer Diagnosis, Prognosis, and Treatment

**DOI:** 10.3390/cancers14030732

**Published:** 2022-01-30

**Authors:** Ming-Tsung Hsu, Yu-Ke Wang, Yufeng Jane Tseng

**Affiliations:** 1 Genome and Systems Biology Degree Program, College of Life Science, Academia Sinica and National Taiwan University, Taipei 106319, Taiwan; trams@cmdm.csie.ntu.edu.tw; 2 Graduate Institute of Biomedical Electronics and Bioinformatics, College of Electrical Engineering and Computer Science, National Taiwan University, Taipei 106319, Taiwan; cragger1027@cmdm.csie.ntu.edu.tw; 3 Department of Computer Science and Information Engineering, College of Electrical Engineering and Computer Science, National Taiwan University, Taipei 106319, Taiwan

**Keywords:** lung cancer, extracellular vesicle, exosome, exosomal protein, exosomal lipid, biomarker, diagnosis, prognosis, treatment, cancer therapy

## Abstract

**Simple Summary:**

Exosomes (or extracellular vesicles) are known to mediate intercellular communication and to transmit molecular signals between cells. Molecules carried by exosomes have their own molecular roles in affecting surrounding and distant environment, as well as recipient cells. Molecular components of exosomes can be used as cancer biomarkers for diagnosis and prognosis, being promising therapeutic targets for the interruption of cellular signals. Therefore, the understanding of the molecular compositions and their functional indications of exosomes has the potential to help doctors to diagnose and monitor diseases and to allow researchers to design and develop potential targeted therapies. This review aims to provide a comprehensive protein and lipid characterization of lung cancer exosomes and to explore their molecular functions and mechanisms regulating physiological and pathological processes. This organization offers informative insight for lung cancer diagnosis and treatment.

**Abstract:**

Exosomes participate in cell–cell communication by transferring molecular components between cells. Previous studies have shown that exosomal molecules derived from cancer cells and liquid biopsies can serve as biomarkers for cancer diagnosis and prognosis. The exploration of the molecules transferred by lung cancer-derived exosomes can advance the understanding of exosome-mediated signaling pathways and mechanisms. However, the molecular characterization and functional indications of exosomal proteins and lipids have not been comprehensively organized. This review thoroughly collected data concerning exosomal proteins and lipids from various lung cancer samples, including cancer cell lines and cancer patients. As potential diagnostic and prognostic biomarkers, exosomal proteins and lipids are available for clinical use in lung cancer. Potential therapeutic targets are mentioned for the future development of lung cancer therapy. Molecular functions implying their possible roles in exosome-mediated signaling are also discussed. Finally, we emphasized the importance and value of lung cancer stem cell-derived exosomes in lung cancer therapy. In summary, this review presents a comprehensive description of the protein and lipid composition and function of lung cancer-derived exosomes for lung cancer diagnosis, prognosis, and treatment.

## 1. Background

Extracellular vesicles (EVs) and exosomes mediate cell–cell communication within the tumor microenvironment and regulate signaling pathways in their target cells [1,2]. Exosomes play multiple roles in physiological and pathological conditions [3]. Additionally, exosomes contribute to tumor progression, such as angiogenesis, oncogenic reprogramming, and premetastatic niche formation [4]. Exosomes can also modulate immune responses [5]. By carrying nucleic acids, proteins, and lipids to neighboring and distant cells, exosomes can trigger tumor initiation, growth, progression, metastasis, recurrence, and therapy resistance [6,7,8,9,10]. The molecules in exosomes can be used as biomarkers for diagnoses and prognoses in disease treatment [11]. The signaling molecules carried by exosomes regulate physiological and pathological processes in recipient cells, thus making exosomes ideal therapeutic targets for cancer therapy [12,13]. It has been proposed that there are three possible strategies to interrupt exosome-mediated communication, including interfering in the biogenesis and/or release of exosomes, removing exosomes out of circulation, and inhibiting the entry of exosomes into recipient cells [13]. The targeting of key molecules in biogenesis pathways can reduce exosome secretion [13]. Moreover, affinity-based technology by using exosome-binding antibodies and lectins enables the capture and removal of exosomes from the circulation [14]. Furthermore, the inhibition of exosome uptake by recipient cells blocks the crosstalk and transfer of molecular messages between cells [13]. Exosomes can also serve as a drug delivery platform for cancer therapy [15]. The appropriate choices of donor cell types and therapeutic cargo-loading methods (such as electroporation, transfection, or incubation) can enable cell-derived exosomes to deliver therapeutic cargos to their targeting sites via proper targeting peptides modified on exosome surfaces [15]. Moreover, EVs could be potential therapeutics for regenerative medicine and wound healing [16,17]. Exosome-based vaccine is another therapeutic option for the treatment of cancer and infectious diseases [18].

The diagnostic and therapeutic potential of exosomes allows for exosomes to possess multiple clinical applications [19]. Several reviews have discussed the potential applications and importance of EVs and exosomes in the fields of various cancers and tumors (e.g., lung [20], liver [21], pancreatic [22], colorectal [23], gastric [24], kidney [25], bladder [26], prostate [27], breast [28], ovarian [29], cervical [30], head and neck [31], thyroid [32], glioma [33], melanoma [34], and hematological malignancies [35]), neurodegenerative diseases (e.g., Alzheimer’s disease [36], Parkinson’s disease [37], Huntington’s disease [38], and amyotrophic lateral sclerosis [39]), mental disorders [40], cardiovascular diseases [41], diabetes mellitus [42,43], and inflammatory and autoimmune diseases [44]. These numerous studies have provided great insights into the diagnostic and therapeutic applications of exosomes in a wide spectrum of diseases.

According to the World Health Organization (WHO), cancer is one of the leading causes of death, and lung cancer is the most common cause of cancer death, accounting for 1.8 million deaths in 2020 [45,46]. Therefore, it is crucial to study tumorigenesis and the therapeutics used to fight cancers, especially lung cancer. Several studies of exosomes in lung cancer have been discussed from multiple perspectives, including the topics of experimental methods and techniques of isolation and characterization, biogenesis and secretion, molecular composition and function, communicative roles within the tumor microenvironment, effect and impact on tumor progression, and clinical applications in cancer therapy, among other perspectives [20,47,48,49,50,51,52,53,54]. Due to the fact that exosomes are involved in physiological and pathological processes, the difference of molecular composition and/or expression levels between exosomes from patients and healthy individuals could provide valuable information for diagnosing and treating diseases. The discovery of important molecules in exosomes of lung cancer samples could facilitate the development of lung cancer therapy. Many researchers have discovered potential molecules in exosomes as being diagnostic and prognostic biomarkers and therapeutic targets for lung cancer. As the studies of exosomal microRNAs (miRNAs) and long noncoding RNAs (lncRNAs) in lung cancer have been organized in other reviews [55,56,57,58,59,60], this review focuses on the studies of the proteins and lipids in lung cancer-derived exosomes. Although crucial proteins have been highlighted in multiple studies and reviews, a comprehensive exosomal protein list and its potential applications are needed, which can provide an overview of therapeutic possibilities for further investigation and development in lung cancer treatment. The complete protein list in this review is summarized from multiple original papers and reviews, in which part of proteins were highlighted [20,47,48,49,50,51,52,53,54,61,62,63,64,65]. In addition, some minor proteins and multiprotein combinations that may act as potential biomarkers are also included in the protein list to expand the possibilities of early detection and cancer treatment and to improve the accuracy of diagnosis, cancer staging, and prognosis assessment. Moreover, exosomal lipid profiles from lung cancer, which have been less discussed, were included in this review to broaden alternative biomarkers for clinical applications in lung cancer.

Exosomes play an important role in cancer progression, and therefore the identification of crucial molecular targets involved in exosome-mediated cell signaling is expected to facilitate tumor growth and advancement [66]. Moreover, the characterization of molecules (especially proteins and lipids) of exosomes from cell lines and biopsies can advance the understanding of cell–cell communication within the tumor microenvironment. This review presents a comprehensive protein and lipid characterization of exosomes in lung cancer. Additionally, this study provides the current scope of potential diagnostic and prognostic biomarkers and therapeutic targets in lung cancer-derived exosomes. This study will help to establish a detailed overview of exosome-mediated tumor progression and other pathological processes.

## 2. Tumor Microenvironment

Cancer cells are abnormal cells that rapidly grow in an uncontrolled manner. These rapidly dividing and uncontrolled cells eventually form a mass of abnormal tissue and tumors. Tumors, stromal cells, and the extracellular matrix (ECM) form a tumor microenvironment that is suitable for tumor growth. Tumors are heterogeneous because they are composed of several cell types, including cancer cells and cancer stem cells (CSCs) [67]. Cancer cells have limited proliferation ability, but CSCs possess stem cell-like self-renewal and differentiation abilities that can drive cancer cell proliferation and tumor growth [68,69]. Stromal cells, including fibroblasts, pericytes, and endothelial cells, can form connective tissue that supports the structure and function of organs. The ECM is a complex and dynamic network structure that is primarily composed of fibrous proteins and proteoglycans [70]. Fibrous proteins, including collagen, elastin, fibronectin, and laminin, provide tensile strength to support matrix structure and to regulate cell adhesion and migration [70]. Proteoglycans are formed by covalent links between glycosaminoglycan (GAG) chains and core proteins. These molecules have buffering, hydration, and binding functions that enable the ECM to withstand compressive forces and to mediate signal transduction [70]. Abnormal ECM remodeling alters the behavior of cells within the tumor microenvironment, such as cancer cells and stromal cells [71]. ECM anomalies promote cell transformation and facilitate tumor-associated angiogenesis, thus leading to the formation of a tumorigenic niche suitable for cancer progression and metastasis [71].

Cells within the tumor microenvironment can directly engage in crosstalk with each other through cell–cell contact, as well as indirectly through cell-secreted soluble factors and cell-derived vesicles [72]. Direct cell contact allows for small molecules to pass through cell junctions, whereas ligand–receptor recognition facilitates cell–cell interactions to transfer cell signals [73]. Soluble factors (such as cytokines, growth factors, and hormones) constitute a class of signaling proteins or other molecules that are secreted alone or carried by EVs [74]. They target cell surface receptors to regulate the behaviors of target cells. Cell-derived vesicles are essential players in cell–cell communications [75]. Membrane-bound vesicles transport molecular cargo to neighboring cells and/or distant cells to modulate cell behavior and to contribute to preparing a suitable niche for cell growth and migration [75]. Moreover, intercellular communication maintains cellular homeostasis and promotes tumorigenesis and tumor progression [76].

## 3. Exosome Biogenesis, Secretion and Uptake

Exosomes have been reported to be derived from several types of body fluids (e.g., blood, urine, saliva, and breast milk) and cell types (e.g., tumor cells, stem cells, and stromal cells) [77]. Exosomes display round and cup-shaped morphologies with a size range extending from 30 to 150 nm in diameter and a density range extending from 1.13 to 1.19 g/mL [7]. Exosomes originate from endosomal compartments and are formed through several intracellular trafficking steps. Cells internalize membrane components via clathrin- or non-clathrin-mediated endocytic pathways [78]. Molecular components encapsulated in endocytic vesicles are delivered to early endosomes [78]. Via the inward budding of the endosomal membrane, intraluminal vesicles (ILVs) are formed inside late endosomes and eventually form multivesicular bodies (MVBs), which are also called multivesicular endosomes (MVEs) [78]. ILVs are released into the extracellular space when MVBs fuse with the plasma membrane, and the released ILVs are called exosomes [78]. Some MVBs are destined to enter lysosomes, and the cargos of these MVBs are degraded within the lysosome [78].

The formation of ILVs (and subsequently MVBs) is driven by the endosomal sorting complex that is required for transport (ESCRT)-dependent and/or ESCRT-independent mechanisms [7]. The ESCRT machinery comprises proteins that assemble into ESCRT-0, ESCRT-I, ESCRT-II, and ESCRT-III complexes [7]. The ESCRT-0 complex recognizes and segregates ubiquitinated proteins into microdomains in the endosomal membrane, after which it recruits ESCRT-I and ESCRT-II complexes to initiate the invagination of the endosomal membrane that simultaneously takes up cytosolic cargo [7]. The ESCRT-III complex subsequently facilitates the cleavage of the invaginated membrane to form ILVs in endosomes [7]. Finally, the ESCRT machinery is disassembled, and the components are recycled upon interaction with the accessory protein VPS4, which is an AAA-ATPase [7]. In contrast, an ESCRT-independent mechanism has been identified on the basis of observations revealing that ILVs continue to form in MVEs even after the functions of ESCRT components have been inhibited [79]. Ceramide and tetraspanins have been described as participants in membrane budding and ILV formation in an ESCRT-independent mechanism [7].

Cells internalize exosomes via several routes: endocytosis, macropinocytosis, phagocytosis, and membrane fusion [80]. The endocytic pathway is mediated by clathrin, caveolin, and/or lipid rafts and involves interactions between exosomal proteins and cell membrane receptors [80]. Clathrin-mediated endocytosis involves a clathrin-coated vesicle formation process that includes (1) nucleation that defines the sites where endocytic events will occur, (2) cargo recruitment that selects specific cargo molecules, (3) clathrin coat assembly that deforms and bends the plasma membrane, (4) vesicle scission that detaches vesicles from the plasma membrane, (5) clathrin uncoating that triggers the recycling of clathrin components, and (6) fusion of clathrin-uncoated vesicles with endosomes that initiates intracellular signaling [81]. Caveolae are cave-like invaginations rich in caveolins, sphingolipids, and cholesterol, and they form a stable membrane domain through caveolin oligomerization and the association with lipid raft domains [80,82]. Caveolae bud inward to internalize EVs and to carry internalized contents to endosomes or caveosomes [80,82]. Macropinocytosis is an endocytic process in which sheet-like membrane ruffles protrude from the cell surface through the reassembly of actin filaments and engulf extracellular fluid and components, pulling them back into the cytoplasm or enclosing them into macropinosomes [83]. Phagocytosis is a receptor-mediated process in which receptor recognition triggers actin polymerization and myosin contraction that enables the cell membrane to extend over opsonized particles and form a phagocytic cup, which enables the internalization of small and large particles [83]. Direct membrane fusion between exosomes and cells is another cargo entry mechanism in which lipid bilayer membranes contact and get closer together, thus leading to hemifusion stalk formation and fusion pore opening and expansion [84].

## 4. Purification and Characterization of Exosomes

Lung cancer samples can be obtained from cancer cell lines and cancer patients. For cancer cell lines, cell culture medium (serum-free medium or exosome-depleted medium) has been used for exosome extraction. For cancer patients, liquid biopsies, such as blood, urine, and saliva, can be sampled, and plasma or serum can be further separated from blood. Cell-released exosomes can then be extracted from cell culture conditioned medium and patient biopsies. On the basis of their physicochemical and biochemical properties, exosomes can be isolated via ultracentrifugation-based platforms (e.g., differential ultracentrifugation and density gradient ultracentrifugation), size-based platforms (e.g., ultrafiltration and size exclusion chromatography), immunoaffinity-based platforms, polymer-based platforms, and microfluidics-based platforms [85]. Guidelines of minimal requirements have been proposed to evaluate the quality of the samples in order to verify EV and exosome preparations [86]. For particle size and shape characterization, size is commonly measured via dynamic light scattering (DLS) and nanoparticle tracking analysis (NTA) techniques, whereas shape is mostly captured by electron microscopy (EM) and atomic force microscopy (AFM) imaging [87]. Several common protein markers (e.g., CD9, CD63, and CD81) are mostly used for exosome characterization, which is frequently conducted and validated via immunoblot analysis [88].

## 5. Protein Composition of Lung Cancer Exosomes

Exosomes are cell-derived vesicles, which implies that the composition of the exosomes reflects the composition of the cells from which they are derived; therefore, the exosomal protein composition can vary by cell source [89]. The inclusion of various cargos in exosome cargo is not a result of random packaging; instead, it is the result of extensively sorted packaging [90]. Exosomes contain proteins, lipids, and nucleic acids (DNA, mRNA, and noncoding RNA) [8]. Moreover, exosomes comprise heterogeneous populations with distinct molecular compositions, even if they are derived from the same parent cells [91]. The heterogeneity of exosomes arises from parent cell activation status and subcellular origin [91]. Currently, two EV-focused databases (ExoCarta [92] and Vesiclepedia [93]) are available and provide information on the protein, lipid, mRNA, and microRNA contents of exosomes and EVs, which have been obtained from various types of samples, including cells and body fluids. Although outdated, these databases remain excellent resources for studying EV molecular profiles and communication roles.

The composition of exosomal proteins reflects the encapsulation of proteins originating from the cytosol and membranes of donor cells. A common set of proteins is carried in most exosomes, regardless of their cellular origin [94,95,96]. Common exosomal proteins include structural and functional proteins and can be classified into tetraspanins (CD9, CD63, and CD81), MVB formation proteins (ALIX and TSG101), and heat shock proteins (HSPs, including HSP70 and HSP90). In addition, exosomes contain other proteins, such as membrane trafficking proteins (Rab GTPases and annexins), cytoskeletal proteins (actin), and adhesion proteins (integrins). Proteins encapsulated in exosomes are then transferred to recipient cells, where they can perform their protein functions.

In addition to the common set of proteins, certain proteins can be found in cell type-specific exosomes. Omics data provide an overview of molecular profiles, which helps to characterize the molecular functions and mechanisms of the regulation of biological processes. Proteomics enables a comprehensive and systematic search for protein profiles. Furthermore, exosomal protein characterization has been performed with many body fluids and cell types. An extensive proteomic characterization of exosomes released from breast cancer cells (such as MDA-MB-231, MCF-7, BT-474, BT-549, and SKBR3 cells) has led to the identification of proteins related to cancer cell growth, progression, migration, metastasis, and immune evasion [97]. Ji et al. found four metastatic factors (i.e., MET, S100A8, S100A9, and TNC), five signal transduction components (i.e., EFNB2, EGFR, JAG1, SRC, and TNIK), and other lipid raft-related components (such as CAV1, FLOT1, FLOT2, and PROM1) that were selectively enriched in exosomes released from human metastatic colorectal cancer cells (SW620 cells), compared to exosomes released from primary colorectal cancer cells (SW480 cells) [98]. Welton et al. showed that exosomes released from human bladder cancer cells (HT1376 cells) have an overrepresentation of proteins related to neoplastic diseases and carcinomas, and 72 of the 353 identified proteins had not been identified in previous studies that were included in the ExoCarta database [99]. Duijvesz et al. identified four proteins (i.e., PDCD6IP, FASN, XPO1, and ENO1) as candidate biomarkers for prostate cancer and showed that they are significantly abundant in exosomes released from human prostate cancer cell lines (PC346C and VCaP cells), compared to exosomes released from prostate epithelial cells (PNT2C2 and RWPE-1 cells) [100]. Liang et al. identified both common and unique sets of proteins in exosomes released from two different human ovarian cancer cell lines (OVCAR-3 and IGROV1 cells), and some of these proteins had not been reported in previous studies that were included in the ExoCarta database [101]. Anderson et al. conducted a proteomic analysis of mesenchymal stem cell-derived exosomes and showed that proteins in the nuclear factor-kappa B (NF-κB) signaling pathway are key paracrine effectors of angiogenesis [102]. Moreover, Grange et al. and Lindoso et al. showed that exosomes released from renal CSCs can promote tumor growth and progression, angiogenesis, and premetastatic niche formation [103,104].

Several exosomal proteins of lung cancer have also been characterized. We summarized the proteins that were identified in exosomes from lung cancer cell lines and patient biopsies, including plasma, serum, urine, saliva, bronchoalveolar lavage (BAL) fluid, pleural effusion, pericardial effusion, and tumor tissue. We not only included all of the major proteins identified in the original literatures but also highlighted some minor proteins mentioned in their original papers. Minor proteins could also be potential biomarkers for lung cancer. Moreover, multimarker combinations are valuable for classification prediction and could be options in addition to single markers for cancer diagnosis. Exosomal proteins and protein combinations are comprehensively organized to display their sample sources and potential applications for lung cancer (Table 1). The significance and value of these proteins as potential biomarkers are elaborated upon as well.

Fourteen proteins (annexin II, CD18, CD54, CD63, CD71, CD80, CD86, CD107a, HSP60, HSC70, HSP70, HSP90, MHC-I, and MHC-II) were expressed in exosomes of the mouse lung cell line 3LL (Lewis lung carcinoma) with and without heat-stressed treatments [105]. Heat stress could stimulate 3LL cells to increase the release of exosomes. When compared to exosomes from tumor cells without heat stress (TEX), heat-stressed tumor cell-derived exosomes (HS-TEX) had more chemokines (CCL2, CCL3, CCL4, CCL5, and CCL20), HSPs (HSP60, HSC70, HSP70, and HSP90), and adhesion molecules (e.g., CD54 and CD86). This chemokine-enriched HS-TEX was more potent than TEX in chemoattracting and activating dendritic cells (DCs) and T cells. Additionally, CD54 was found to be one of the molecules involved in the adhesion of exosomes to DCs, whereas MHC-I or MHC-II presented by exosomes may be responsible for the activation of T cells. HS-TEX was suggested to be an efficient tumor vaccine to elicit antitumor immunity. Moreover, Hsp72 and Hsc73 were expressed in exosomes from a human cell line (H23 lung AC) and in exosomes from 3 mouse cell lines (EL4 thymoma, TS/A mammary carcinoma, and CT26 colon carcinoma) [106]. Myeloid-derived suppressor cells (MDSCs) are immature myeloid cells with the immunosuppressive ability to suppress T cell activation. Hsp72 found on the surfaces of tumor-derived exosomes could mediate MDSC activation, thus leading to the inhibition of T cell proliferation, which may contribute to cancer development. The mechanism of Hsp72-induced Stat3 phosphorylation in MDSCs was observed to be dependent on the autocrine production of IL-6 and the TLR2/MyD88 signaling pathway. Additionally, HSP70 was observed to be highly expressed on exosomes from the A549 lung cancer cell line [107]. A549 cell-derived exosomes have been found to stimulate the secretion of inflammatory cytokines (IL-6, IL-8, and MCP-1) in mesenchymal stem cells (MSCs), which can educate MSCs into proinflammatory MSCs (P-MSCs). This process was found to be induced by the exosomal surface protein HSP70 through the activation of the TLR2/NFκB signaling pathway. Moreover, P-MSCs have been found to have an enhanced capacity to recruit macrophages and to promote tumor growth. In both breast and lung cancer patients, the levels of HSP70-positive exosomes were observed to be higher in metastatic patients than in nonmetastatic patients and healthy individuals [108]. Furthermore, HSP70-positive exosomes had a better ability than circulating tumor cells (CTCs) in distinguishing metastatic patients from nonmetastatic patients. In addition, exosomal HSP70 levels have been observed to be correlated with HSP70 expression in tumor biopsies. Their follow-up case studies showed that the change in exosomal HSP70 concentration in the plasma of cancer patients was associated with disease status and inversely correlated with treatment responses, thus indicating that exosomal HSP70 levels could be utilized to monitor tumor progression and the response to cancer therapy.

EGFR was found in microvesicles (MVs) from the human cancer cell line A431 (skin epidermoid carcinoma), as well as from A459 (lung carcinoma) and DLD-1 (colorectal carcinoma) cell lines [109]. Through phosphatidylserine (PS) and intact EGFR kinase activity presented by MVs, EGFR-positive MVs could transmit cancer cell-derived EGFR signals to activate MAPK and Akt pathways in human umbilical vein endothelial cells (HUVECs). Oncogenic EGFR signaling could also trigger the expression of vascular endothelial growth factor (VEGF) and autocrine signaling of VEGF receptor-2 (VEGFR-2) in HUVECs. In addition, a tumor xenograft mouse model suggested that tumor-derived MVs could transfer EGFR to tumor blood vessels and contribute to tumor growth and angiogenesis. EGFR has been detected in exosomes isolated from fresh lung biopsies of non-small cell lung cancer (NSCLC) patients [110]. EGFR was also found to be positive in more than 80% of exosomes from NSCLC patients and in less than 2% of exosomes from chronic lung inflammation patients. Moreover, EGFR-positive exosomes were shown to have the ability to drive DCs to generate tolerogenic DCs that can induce tumor-specific regulatory T cells. EGFR has been detected in exosomes from cell culture supernatants of five human cancer cell lines (HARA, HARA-B, A549, RERF-LC-MS, and LU65), the plasma of HARA-B tumor-bearing mice, and the plasma of human lung cancer patients [111]. The expression levels of EGFR in cell-derived exosomes were found to be concordant with those in their original cells. Exosomal EGFR levels in the plasma of xenograft mice showed the same trend as the size of xenograft tumors, thus implying that exosomal EGFR levels could be utilized to estimate tumor size. When comparing lung cancer patients to healthy individuals, the levels of exosomal EGFR were significant higher in five of nine lung cancer patients, while the levels of soluble EGFR in plasma were not significantly different in seven of nine lung cancer patients. Additionally, EGFR in plasma exosomes has been suggested to be a possible biomarker for lung cancer diagnosis. Moreover, an innovative nanoparticle-based biomaterial was developed for eliminating circulating biohazards out of the body [112]. Mesoporous silica nanoparticles (MSNs) coupled with EGFR-targeting aptamers (MSN-AP) were designed for capturing circulating exosomes in the blood. The aptamer-coupled nanoparticle MSN-AP can recognize and bind to EGFR-positive exosomes. Flow cytometry analysis has shown that exosomes from the human lung cancer cell line A549 (A-Exo) had higher EGFR expression than exosomes from the human embryonic lung fibroblast HELF (H-Exo). The bound conjugate between MSN-AP and A-Exo (MSN-Exo) could transverse across liver cells via endocytosis and exocytosis. Further evidence has shown that MSN-AP could accelerate the hepatobiliary excretion of A-Exo into the small intestine, and attenuate A-Exo-induced metastasis. Moreover, a late stage NSCLC patient had more targeted EGFR-positive exosomes in the blood than early stage patients, thus implying that the number of EGFR-positive exosomes may be correlated with the stage of lung cancer.

AREG has been shown to be enriched in exosomes from two lung cancer cell lines (CRL-2868 and A549) and enriched in exosomes from a prostate cancer cell line (PC3) and a breast cancer cell line (MDA-MB-231), compared to their original cells [113]. AREG has also been detected in exosomes from plasma samples of NSCLC patients with different disease stages. Additionally, CRL-2868 cell-derived exosomes have been shown to activate the EGFR pathway and induced the expression of RANKL and osteoclastogenesis markers (TRAP and MMP9) in preosteoclasts. Evidence has shown that exosomal AREG is an important protein in this process and promotes osteoclast differentiation, thus leading to osteolytic bone metastasis. Similar results were also confirmed for exosomes of the A549 cell line and for NSCLC patients.

Eighteen proteins (BAIP2, CPNE1, FIBA, CO5, CD91/LRP1, MASP1, APOB, CO8A, PLMN, PGBM, PERM, FCGBP, ITA2B, KV313, IC1, A2MG, CD317/BST2, and HABP2) have been identified in exosomes from the serum samples of lung cancer patients, including adenocarcinoma (AC) and squamous cell carcinoma (SCC) [114]. On the basis of the exosome sandwich enzyme-linked immunosorbent assay (ELISA) system, researchers demonstrated exosomal CD91 to have an early detection power for lung AC but not for lung SCC. Furthermore, the combination of this exosomal biomarker CD91 and one clinical biomarker, known as carcinoembryonic antigen (CEA), improved the diagnostic ability of distinguishing lung AC patients from healthy individuals and interstitial pneumonia patients.

EV arrays, which represent a new application of protein microarrays printed by customized capturing antibodies, have been developed to capture and detect exosomes from unpurified samples, including plasma and cell culture supernatants [147]. EV arrays can phenotype and semiquantify exosomes on the basis of a small quantity of samples. Several clinical applications of EV arrays have been conducted to explore exosomal proteins as diagnostic and prognostic biomarkers in lung cancer [115,116,117]. Thirty-seven proteins were adopted in an EV array for detecting exosomal proteins in plasma samples from lung AC patients (IIIa-IV) [115]. A 30-marker model showed diagnostic potential with the largest area under the curve (AUC), which is a metric for evaluating the performance of classification and diagnosis [148]; additionally, the model showed a 75.3% accuracy for the classification between the advanced NSCLC patient group and the noncancer patient group. Eighteen exosomal proteins (CD81, TAG72, CD63, TSG101, CD9, CD163, MUC1, N-cadherin, EGFRvIII, c-MET, flotillin-1, HB-EGF, EGFR, TNF RI, NY-ESO-1, CD146, CD142, and PLAP) were involved in the 30-marker panel, which was composed of 11 markers (CD81, MUC1, CD63, HB-EGF, EGFR, N-cadherin, NY-ESO-1, EGFRvIII, TAG72, CD142, and PLAP), 3 markers with normalized values (*CD9, *CD81, and *CD63), and 16 marker relations (TAG72/*CD63, CD63/TAG72, TSG101/*CD63, CD163/*CD63, CD63/EGFRvIII, N-cadherin/*CD63, EGFRvIII/*CD63, c-MET/EGFRvIII, TAG72/c-MET, MUC1/*CD63, CD63/flotillin-1, EGFRvIII/*CD81, CD81/EGFRvIII, TNF RI/*CD63, EGFRvIII/*CD9, and CD146/*CD63). The 11 markers are the protein signals (log2-transformed) on the EV array; markers with normalized values (marked by “*”) are the protein signals normalized by total signals on the EV array (i.e., the percentage of each protein signal); and marker relations (marked by “/”) are the individual relations between protein signals on the EV array. A 49 antibody-printed EV array was used to profile exosomal proteins in the plasma of lung cancer (AC, SCC, and SCLC) patients [116]. When compared to the cohort of patients without cancer but who had other lung symptomatic diseases, CD151 was significantly upregulated in exosomes from cohorts of AC patients, SCC patients, SCLC patients, and patients with all three subtypes. Exosomal CD151 could be a biomarker for distinguishing lung cancer patients with every subtype from noncancer patients. CD171 was only significantly upregulated in exosomes from the AC group, whereas TSPAN8 was significantly upregulated in both AC and SCC group-derived exosomes. Both exosomal CD171 and TSPAN8 could be used to separate lung AC patients from noncancer patients. CD151, CD171, and TSPAN8 were concluded to be promising exosomal biomarkers for distinguishing patients with lung cancer from patients without cancer. Moreover, the combination of 10 markers (CD151, TSPAN8, NY-ESO-1, HER2, CD171, EGFRvIII, SFTPD, flotillin-1, CD142, and mucin16) showed the largest AUC performance for the discrimination between lung cancer with all subtypes and noncancer, whereas the model with another combination of 10 markers (CD151, TSPAN8, CD171, SFTPD, CD82, PLAP, NY-ESO-1, HER2, flotillin-1, and mucin16) had the largest AUC value for the classification of lung patients with the AC subtype. This implied that these markers were also present in exosomes of lung cancer patients, although some of them (flotillin-1, NY-ESO-1, CD82, mucin16, and PLAP) presented higher signal intensities in exosomes from the noncancer group. Another EV array application demonstrated that proteins in NSCLC plasma exosomes can be promising prognostic biomarkers for NSCLC [117]. According to the presence of exosomal proteins, four proteins (CD171, flotillin-1, HER3, and GRP78) were found to be potential markers to predict superior survival of NSCLC patients; nevertheless, CD171 was the most likely biomarker for the prediction. According to the expression levels of exosomal proteins, seven proteins (NY-ESO-1, EpCam, CAIX, CD13, EGFR, PLAP, and CD276) that exhibited increasing levels displayed a significant prognostic potential for NSCLC. Three out of the seven proteins (NY-ESO-1, EGFR, and PLAP) had a significant predictive ability to predict inferior survival, and NY-ESO-1 showed a strong association with inferior survival. Due to the fact that most of the exosomal proteins were only detectable in a subset of the patient cohort, only patients with measurable levels were included for further analyses. According to exosomal protein levels of the patient subset, nine proteins (NY-ESO-1, HER3, CAIX, EpCam, CD13, PLAP, CD276, EGFR, and Alix) exhibited a significant value for overall survival (OS) prediction of NSCLC. Five out of the nine proteins (NY-ESO-1, EpCam, PLAP, EGFR, and Alix) had significant prognostic potential for predicting inferior survival, and only NY-ESO-1 was a strong biomarker for inferior OS prediction. NY-ESO-1 was finally concluded to be a significant exosome-bound prognostic biomarker for inferior survival prediction of NSCLC, although the other proteins were still valuable and warrant for further investigation.

A proteomic analysis showed the potential to discover diagnostic and prognostic biomarkers for lung cancer [118,119,120,121,122,123,124,125]. Via a proteomic analysis, 912 proteins were identified in MVs from pleural effusion samples of three NSCLC patients, including several tumor-associated proteins such as EGFR, KRAS, BSG/EMMPRIN/CD147, CEACAM6, CLDN1, CLDN3, and RAB family proteins (e.g., Rab1, Rab3, Rab5, Rab6, Rab11, and Rab13) [118]. A total of 523 of 912 MV proteins were in at least two patients, and 318 MV proteins were in all three patients. Other proteins such as tetraspanins, RAS proteins (e.g., NRAS and RRAS), MHC proteins, complement component proteins, Src family kinases (e.g., SRC, LYN, HCK, and FGR), S100 calcium-binding proteins, and 14-3-3 proteins were found in the pleural effusion-derived MVs. HSP90, Src, Rac, ERK2/MAPK1, and CAV1 in MVs were confirmed via a Western blot analysis. For comparison with other body fluids (i.e., plasma/serum, BAL, and pleural effusion), 153 out of 912 MV proteins were pleural effusion-specific proteins, including ABI1, BSG, CAV1, GRB2, RAS proteins, and SRC. According to lung protein annotation data in the HPRD (Human Protein Reference Database: https://www.hprd.org/) (accessed on 27 December 2021), Park et al. found 264 MV proteins were derived from lung tissue, and 67 out of them were commonly identified in all three patients, including ANXA4, AQP1, RAS proteins, and CEACAM6. This study explored a pool of MV proteins and suggested that they could be diagnostic biomarkers and therapeutic targets. Several proteins were identified to be significantly enriched in exosomes from A549 cells (NSCLC cell line) and HCC827 cells (NSCLC cell line), compared to exosomes from HBE4 cells (immortalized normal human bronchial epithelial cell line) [119]. Proteins enriched in both A549 and HCC827 exosomes included exosome markers and associated proteins (PDCD6IP, TSG101, CD63, CD9, and SDCBP), cell adhesion proteins (DSG2, CD151, and CNTN1), receptors and signaling proteins (EGFR, SRC, EDIL3, ITGB6, JAK1, and GRB2), NSCLC-related proteins (ITGB1, BSG, SLC3A2, LAMP2, and CEACAM6), and proteases (PAPPA, CTSA, and GGT3P). CD81, extracellular matrix proteins (MFGE8 and HSPG2), and receptors (GPRC5A) were enriched in A549 exosomes, whereas SDC1, cell adhesion proteins (EPCAM, MPZL1, and MPZL2), receptors and signal transduction molecules (TACSTD2, CTNNB1, and RALA), and enzyme modulators (GNB1 and TOM1L1) were enriched in HCC827 exosomes. This quantitative proteomic study identified several exosomal protein candidates that could be further explored to develop single or multiple biomarkers for NSCLC diagnosis. An affinity chromatography column combined with a filter system (ACCF) was developed to prepare EVs from viscous saliva samples with highly abundant proteins [120]. Via the ACCF system, 63 proteins were uniquely identified in salivary EVs of lung cancer patients, whereas 50 EV proteins were commonly identified in both patient and healthy individual samples. Twenty-five of the 63 unique proteins were known to participate in the cancer-related network. Among the 25 cancer-related EV proteins, 12 proteins (annexins (A1, A2, A3, A5, A6, and A11), NPRL2, CEACAM1, MUC1, PROM1, HIST1H4A, and TNFAIP3) were related to lung cancer, according to their literature survey. Exosomal proteins of saliva and serum samples from lung cancer patients and healthy individuals were identified and quantified via label-free quantification [121]. Comparisons between subject types (i.e., lung cancer patients and healthy individuals) identified 86 potential exosomal proteins for lung cancer detection. Eleven of the 86 exosomal proteins were further identified as unique proteins in lung cancer, on the basis of comparisons between sample types (i.e., saliva and serum). By applying different data analysis strategies to proteomic data, researchers found that 11 proteins in both saliva and serum exosomes were highly potent candidates for lung cancer diagnosis, among which A1AG1, AQP5, and MUC5B were highlighted due to their association with cancer. MUC5B, IQGAP, ENO1, and SPARCL1 were identified in salivary exosomes of lung cancer patients from a comparative proteomics analysis and confirmed via a Western blot analysis [122]. When lung cancer subjects were compared with normal subjects, the protein expression levels of MUC5B and IQGAP in salivary exosome samples were significantly higher in the lung cancer group; however, none of the four protein expression levels in saliva samples showed a significant difference. This study presented the value of exploring and using potential protein biomarkers in exosomes from noninvasive saliva samples for lung cancer diagnosis. Small extracellular vesicles (sEVs) from two human lung carcinoma cell lines (95C (poorly metastatic) and 95D (highly metastatic)) were used to explore metastasis-related proteins [123]. Proteins with differential abundance in 95C-derived sEVs and 95D-derived sEVs were identified via a quantitative proteomics analysis, and 62 differential proteins were found to be involved in the pathway of cell movement via a pathway analysis, among which HGF was the most upregulated protein in sEVs of a highly metastatic cell line (such as 95D). When compared to 95C xenograft mice, 95D xenograft mice had higher HGF expression in plasma sEVs. When compared to healthy individuals and lung cancer patients with early stage (I/II) disease, lung cancer patients with late stage (III/IV) disease had enriched HGF in their plasma sEVs. Via the TCGA (The Cancer Genome Atlas: https://tcga-data.nci.nih.gov/) (accessed on 27 December 2021) data analysis, an OS prediction suggested that HGF is a promising biomarker for late stage cancer or poor prognosis. Further experiments showed that sEV-HGF could activate the HGF/c-Met signaling pathway to promote cancer cell metastasis. Exosomes released from A549 lung cancer cells under four treatment conditions (normoxia, normoxia irradiation, hypoxia, and hypoxia irradiation) had different degrees of impact on target cells (i.e., A549 cells and HUVECs in their study) [124]. When compared to negative control, migratory and invasive abilities of A549 cells were raised sequentially by exosomes from hypoxic irradiated cells, hypoxic cells, normoxic irradiated cells, and normoxic cells. In addition, A549-derived exosomes could induce tube formation of HUVECs. Hypoxia-induced exosomes could promote A549 and HUVEC proliferation, and irradiation effects under both normoxia and hypoxia conditions could further enhance their cell proliferation abilities. When mass spectrometry (MS) data of exosomes from different conditions were compared, exosomal proteins ACLY, ANGPTL4, LRP1, PSMD14, TKT, TTN, and VCAN were significantly upregulated under the hypoxia irradiation condition. Among them, ANGPTL4 had the most abundant expression level. An A549 cell line with ANGPTL4 knockdown was established for verifying the roles of ANGPTL4. Under the same treatment condition, exosomes from ANGPTL4-knockdown A549 cells had weakened effects on migratory ability of A549 cells, compared to exosomes from A549 cells without ANGPTL4 knockdown. Mo et al. suggested that ANGPTL4 could be a diagnostic biomarker for cancer metastasis and a therapeutic target for lung cancer. Proteomic profiling of extracellular vesicles and particles (EVPs) has been conducted in 426 human samples (including 274 tumor and 152 nontumor samples) from different sample sources (including cell lines, tissue explants, blood plasma and serum, bone marrow, lymphatic fluid, and bile duct fluid specimens) [125]. Cancer patient-resected tissue and plasma samples from multiple cancers (including pancreatic, lung, breast, colorectal carcinomas, melanoma, neuroblastoma, and osteosarcoma) were further analyzed. In tissue samples from lung cancer, 123 EVP proteins were shown to be present in at least half of 14 lung AC samples and to have signals in tumor samples at least 10-fold higher than signals in nontumor samples. The top 30 EVP proteins are FHL2, XRN2, GLRX, HDLBP, SRRT, RCC1, AP3S1, SNRPD3, NOP2, RPL22, DNAJC7, STK39, SRP54, DHX36, ELAVL1, THBS2, ACO2, ACBD3, SRP9, THOC2, HNRNPC, EIF5B, RALY, UCHL5, KHDRBS1, SF3B6, WDR44, BABAM2, HTATIP2, and CSTF3. Additionally, 2 of the 123 EVP proteins (HTATIP2 and METTL1) were found only in the tumor tissue (TT) but not in the matched nontumor adjacent tissue (AT) and distant tissue (DT). Taken together, HTATIP2 is the one in the top 30 protein list and the one only in the TT, thus implying that HTATIP2 has the potential as a lung tumor-specific biomarker. In plasma samples from lung cancer, RHOV and CLDN5 have been shown to be present in samples from more than 30% of 12 lung AC patients, but not in samples from all 28 healthy individuals. RHOV was further validated to have higher expression levels in plasma-derived EVPs from lung AC patients, compared to EVPs from healthy individuals.

TGF-β and IL-10 were expressed in exosomes of NCI-H1688 cells (SCLC cell line) and NCI-H2228 cells (NSCLC cell line) [126]. When compared to exosomes of HEK 293T cells (a human embryonic kidney cell line), the concentration levels of TGF-β and IL-10 were increased in exosomes of the two human lung cancer cell lines, particularly in exosomes from cells cultured under hypoxic conditions. Both TGF-β and IL-10 are essential factors that promote lung cancer cell migration.

VIM, an intermediate filament protein, has been found in exosomes from PC14 (nonmetastatic) and PC14HM (highly metastatic) lung cancer cell lines [127]. Both the mRNA and protein expression levels of VIM in PC14HM-derived exosomes were significantly higher than that in PC14-derived exosomes. Moreover, the mRNA expression of VIM in serum-derived exosomes from late stage lung cancer patients was significantly higher than that in exosomes from healthy individuals and early stage lung cancer patients. Exosomes from the highly metastatic cell line (i.e., PC14HM cells) and the late stage cancer patients had higher abilities to induce migration, invasion, and proliferation of the recipient human bronchial epithelial cells (HBECs). Additionally, HBECs treated by highly metastatic cell-derived exosomes and late stage cancer serum-derived exosomes had higher VIM expression compared to HBECs treated by nonmetastatic cell-derived exosomes and healthy serum-derived exosomes, respectively. The knockdown of VIM in late stage cancer serum-derived exosomes demonstrated that the migratory ability of HBECs treated by the VIM depleted exosomes was reduced, and the VIM expression in the treated HBECs was also decreased. The above evidence suggested that lung cancer-derived exosomal VIM may mediate cell migration and epithelial–mesenchymal transition (EMT) of the noncancerous recipient cells (i.e., HBECs).

Thirteen proteins (SRGN, TPM3, THBS1, HUWE1, CCDC18, ALDH1L1, HIST1H4A, NCCRP1, MED14, BHMT, GLUD1, PPIA, and EXOC8) were enriched in plasma-derived EVs of lung AC patients, with concordant expression in EVs of lung AC cell lines (H23, H647, H1573, and HCC4019) [128]. The combination of SRGN, TPM3, THBS1, and HUWE1 was further demonstrated via receiver operating characteristic (ROC) curve analyses to yield an AUC of 0.8995.

Tim-3 and galectin-9 have shown significantly elevated expression in plasma-derived exosomes of NSCLC patients (compared to healthy individuals) and lung SCC patients (compared to lung AC patients) [129]. Both exosomal Tim-3 and galectin-9 expression have been found to be positively correlated with clinicopathological features, including patient age, tumor size, distant metastasis, and cancer stage. In addition, exosomal Tim-3 was also associated with more lymph node metastasis. Therefore, exosomal Tim-3 and galectin-9 could be potential biomarkers for clinical application in NSCLC.

The expression of LBP in serum-derived exosomes from NSCLC patients was observed to be significantly higher than that in exosomes from healthy individuals [130]. Further analyses showed that patients with metastatic NSCLC had higher exosomal LBP expression than patients with nonmetastatic NSCLC, whereas the difference between patients with early stage (I to IIIa) and healthy individuals was not significant. Evidence has shown that exosomal LBP could be a promising metastatic biomarker for distinguishing metastatic NSCLC patients from nonmetastatic NSCLC patients.

Both AHSG and ECM1 have been shown to have significantly higher expression in serum-derived exosomes of NSCLC patients than in those of healthy individuals and were proposed to be diagnostic biomarkers for NSCLC [131]. The diagnostic ability of AHSG and ECM1 was evaluated via ROC curves and AUC values for distinguishing all stage (I-IV) patients from healthy individuals and for distinguishing early stage (I-IIA) patients from healthy individuals. Their results revealed that the combination of AHSG and ECM1 has a better diagnostic ability than the abilities of individual markers. The diagnostic ability was even improved when combining one or two of the proposed exosomal biomarkers (AHSG and ECM1) with the serum tumor biomarker CEA.

Nineteen proteins (THBS1, ANXA6, HIST1H4A, COL18A1, MDK, CD151, SRGN, ENO1, TUBA4A, SLC3A2, GPI, MIF, MUC1, TALDO1, SLC7A5, ICAM1, HSP90AA1, G6PD, and LRP1) were more than fivefold more highly expressed in exosomes of NCI-H838 cells (NSCLC cell line) than in the cellular membrane of NCI-H838 cells [132]. These proteins have been recorded as potential biomarkers for lung cancer diagnosis. Further analyses showed that MUC1 is significantly upregulated in plasma-derived exosomes of NSCLC patients, and it has been suggested that exosomal MUC1 may be a valuable diagnostic biomarker to distinguish NSCLC patients from healthy individuals.

Integrins α3 and β1 have been shown to be expressed in exosomes from four lung AC cell lines (A549, H1975, H3255, and H1650) and one NSCLC patient pericardial effusion sample [133]. This study demonstrated that α3β1 integrin-expressing cells and exosomes could be targeted by the cancer-targeting peptide LXY30. LXY30 could detect metastatic tumors and may be an attractive platform for the targeted delivery of cancer therapeutics, thus implying that the target α3β1 integrin could be used with integrin-targeting peptides for cancer diagnosis.

LRG1 has been detected in exosomes from urine samples of NSCLC patients without chemotherapy [134]. The proportion of LRG1 expression in lung tumor tissues was significantly higher than that in adjacent nontumor lung tissues, thus implying that LRG1-positive exosomes may be derived from lung tumor tissues. Additionally, LRG1 expression in urinary exosomes of NSCLC patients was significantly increased in comparison with that of healthy individuals, thus suggesting that LRG1 could be a potential urinary biomarker for noninvasive NSCLC diagnosis. Both the mRNA and protein levels of LRG1 were enriched in exosomes from NSCLC cell lines (SPCA1, A549, PC9, H1299, and H358) compared to those from normal HBECs (BEAS-2B) [135]. In addition, LRG1 expression levels in plasma-derived exosomes of NSCLC patients were higher than those of healthy individuals. Among these NSCLC patients, patients with lymph node metastasis had higher exosomal LRG1 expression than patients without lymph node metastasis. Further evidence showed that LRG1 transferred by A549-derived exosomes can promote the angiogenesis of HUVECs via the TGF-β pathway.

The protein expressions of HLA-class I, BAGE, PD-L1, and annexin-A2 have been shown to be elevated in exosomes from BAL fluids of smokers and NSCLC patients, compared to the expression in exosomes of healthy individuals [136]. In their study, some differentially expressed miRNAs, mRNAs, and lncRNAs were also found in exosomes from smokers and NSCLC patients. Therefore, smoking contributes to the dysregulation of exosomal molecule expression, and the dysregulation may be involved in lung cancer development.

PD-L1 has been found in exosomes from melanoma cells, as well as from lung (H1299, H358, and H1264) and breast (MDA-MB-231) cancer cells [137]. Moreover, the expression of exosomal PD-L1 is upregulated by interferon-γ (IFN-γ) [137], which is a cytokine secreted by numerous immune cells [149]. The interaction between PD-L1 on exosomes and programmed death-1 (PD-1) on T cells enables tumor cells to suppress T cell-mediated antitumor immunity [137]. Immunosuppressive signals could help tumor cells to escape immune surveillance and facilitate tumor growth. PD-L1 has also been detected in exosomes from NSCLC cell lines (A549, H460, and H1975) and plasma samples of NSCLC patients [138]. The expression levels of PD-L1 in cell-derived exosomes were found to be proportional to those in their original cell lines, whereas the percentage of PD-L1-positive exosomes from each patient was correlated with the expression level of PD-L1 in its tumor tissue. PD-L1 expression in plasma-derived exosomes from advanced NSCLC patients before and after 2 months of immune checkpoint inhibitor (ICI) treatment was previously measured [139]. Patients with a fold change of exosomal PD-L1 greater than or equal to 1.86 had better progression-free survival (PFS) and OS rates. Therefore, increased exosomal PD-L1 expression could be used as a biomarker for treatment efficacy and OS prediction of advanced NSCLC. PD-L1 was also expressed in serum-derived exosomes of NSCLC patients [140,141]. The concentration of exosomal PD-L1 was found to be higher in patients than in healthy individuals [140]. Exosomal PD-L1 levels have also been found to be significantly correlated with four clinicopathological characteristics (TNM stage, tumor size, lymph node status, and distant metastasis) [140], tumor PD-L1 levels, and the number of CD8-positive tumor-infiltrating lymphocytes [141]. A single molecule array (Simoa) immunoassay has been designed for capturing and detecting EVs from lung cancer cells (A549 and SK-MES1) and patients [142]. EVs were first captured by anti-Epcam antibody-coated magnetic beads, and PD-L1-positive EVs were subsequently captured by biotinylated PD-L1 detection antibody and detected by the released fluorescent signals. Simoa Epcam-PD-L1 signals from tumor-derived EVs have been shown to be significantly correlated with tumor tissue PD-L1 expression levels. The Simoa immunoassay may be an alternative platform to measure PD-L1 expression on circulating tumor-derived EVs for diagnosing PD-L1-positive patients and monitoring therapeutic responses. Another study adopted a CellSearch platform to enrich EpCAM-positive EVs and two customized Simoa immunoassays to measure PD-L1 levels [143]. Lung (i.e., A549 and H1975 cell lines and NSCLC patients) and breast (i.e., MDA-MB-231 cell line and triple-negative breast cancer (TNBC) patients) samples were prepared for the isolation of total EVs (via differential ultracentrifugation) and EpCAM-positive EVs (via anti-EpCAM magnetic nanoparticles in CellSearch platform). Simoa immunoassays showed that PD-L1 was observed in total EVs from plasma samples of cancer patients and healthy individuals. However, PD-L1 was only observed in EpCAM-positive EVs from cancer patient samples, thus suggesting that tumor-derived EVs could be enriched by the CellSearch platform.

The expression levels of seven proteins (CD5L, CLEC3B, SERFINF1, ITIH4, SAA4, SERFINC1, and C20ORF3 (APMAP)) were found to be significantly increased in serum-derived EVs of lung cancer patients, including AC, SCC, and small cell lung cancer (SCLC) [144]. Further results showed that only CD5L is significantly upregulated in cancer tissues, which suggested that CD5L can be an effective biomarker for lung cancer diagnosis. Although the other six proteins did not show significant differences between cancer and normal tissues, they were still proposed to be potential biomarkers because of their higher expression in EVs of lung cancer patients.

TUBA1C, GAPDH, KRT25, GCC2, and POTEKP were collectively identified in exosomes from five NSCLC cell lines, including A549 (with the *KRAS* mutation), H1299 (with the neuroblastoma *RAS* (*NRAS*) mutation), PC9 (with the *EGFR* mutation), H1650 (with the *EGFR* mutation), and H522 (with the *TP53* mutation), but they were not identified in exosomes from human pulmonary alveolar epithelial cells (HPAEpiCs), thus indicating that the five proteins were lung cancer exosome-specific proteins [145]. RNA and protein expressions of exosomal GCC2 were further verified, and both levels in H1299 and H522 exosomes were higher than the levels in HPAEpiC exosomes. GCC2 was also detected in plasma-derived exosomes of AC patients, and the protein levels in patients were significantly higher than the levels in healthy individuals. Moreover, exosomal GCC2 protein levels gradually increased during cancer progression. Therefore, exosomal GCC2 protein expression could distinguish early stage patients from healthy individuals, thus suggesting that exosomal GCC2 could be used for the early diagnosis of lung cancer.

Both RNA and protein of exosomal eIF4E (exo-eIF4E) were detected in serum samples from NSCLC patients and healthy individuals [146]. RNA expression of exo-eIF4E in NSCLC patients was significantly higher than that in healthy individuals. Moreover, exo-eIF4E RNA expression in late stage (III + IV) patients was significantly higher than that in early stage (I + II) patients, thus suggesting RNA expression of exo-eIF4E may correlate with cancer progression. RNA level of exo-eIF4E was associated with some clinicopathological features, including TNM stage, distant metastasis, and the level of CYFRA21-1, thus implying that exo-eIF4E could be a prognostic biomarker for NSCLC patients. Exo-eIF4E RNA level could be an independent indicator to predict OS and PFS of NSCLC patients. Although this study mainly focused on RNA level, it may be worth further investigating the protein level as eIF4E protein was also detected in the tumor-derived exosomes.

**Figure 1:**
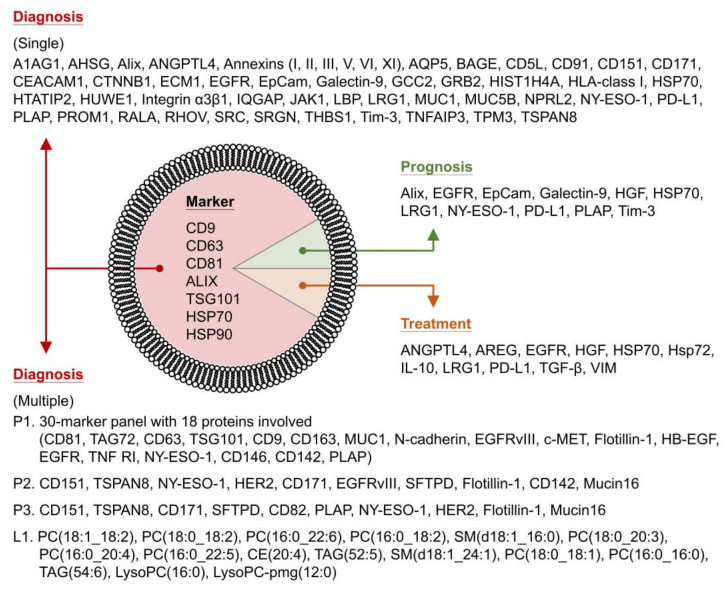
Potential biomarkers for lung cancer diagnosis, prognosis, and treatment. Biomarkers are exosomal proteins and lipids of lung cancer samples. A single marker (A1AG1, AHSG, Alix, ANGPTL4, Annexins (I, II, III, V, VI, XI), AQP5, BAGE, CD5L, CD91, CD151, CD171, CEACAM1, CTNNB1, ECM1, EGFR, EpCam, Galectin-9, GCC2, GRB2, HIST1H4A, HLA-class I, HSP70, HTATIP2, HUWE1, Integrin α3β1, IQGAP, JAK1, LBP, LRG1, MUC1, MUC5B, NPRL2, NY-ESO-1, PD-L1, PLAP, PROM1, RALA, RHOV, SRC, SRGN, THBS1, Tim-3, TNFAIP3, TPM3, and TSPAN8) indicates that a single protein is used for the diagnosis. The set of multiple markers means that the combination of multiple proteins in one single set (P1: CD81, TAG72, CD63, TSG101, CD9, CD163, MUC1, N-cadherin, EGFRvIII, c-MET, Flotillin-1, HB-EGF, EGFR, TNF RI, NY-ESO-1, CD146, CD142, and PLAP; P2: CD151, TSPAN8, NY-ESO-1, HER2, CD171, EGFRvIII, SFTPD, Flotillin-1, CD142, and Mucin16; and P3: CD151, TSPAN8, CD171, SFTPD, CD82, PLAP, NY-ESO-1, HER2, Flotillin-1, and Mucin16) or multiple lipids in one single set (L1: PC(18:1_18:2), PC(18:0_18:2), PC(16:0_22:6), PC(16:0_18:2), SM(d18:1_16:0), PC(18:0_20:3), PC(16:0_20:4), PC(16:0_22:5), CE(20:4), TAG(52:5), SM(d18:1_24:1), PC(18:0_18:1), PC(16:0_16:0), TAG(54:6), LysoPC(16:0), and LysoPC–pmg(12:0)) should be used for the diagnosis. Diagnosis is the discrimination between the lung cancer group (i.e., patients with lung cancer) and the control group (i.e., healthy individuals or patients without lung cancer). Prognosis (Alix, EGFR, EpCam, Galectin-9, HGF, HSP70, LRG1, NY-ESO-1, PD-L1, PLAP, and Tim-3) could involve treatment response monitoring, clinicopathological correlation, and overall survival prediction. Treatment represents some of the mechanisms or pathways of the proteins (ANGPTL4, AREG, EGFR, HGF, HSP70, Hsp72, IL-10, LRG1, PD-L1, TGF-β, and VIM) that have been studied and could be used for further investigation and development for lung cancer therapy (please refer to the section “Protein composition of lung cancer exosomes” and original papers for marker usage).

Proteins in lung cancer-derived exosomes are diagramed to illustrate promising usage as diagnostic and prognostic biomarkers or therapeutic targets for lung cancer therapy (Figure 1).

## 6. Protein Function of Lung Cancer Exosomes

Exosomes perform their communication effects by transmitting their molecular cargo from donor cells to recipient cells [150]. Therefore, the elucidation of the functions of their cargo proteins is helpful in clarifying the roles and effects of exosomes during the intercellular communication process and is crucial for exploring lung cancer cell-mediated signaling processes. Due to the fact that the functions and indications of several proteins that have been reported in various studies indicate the importance of protein molecules in tumor growth, progression, and metastasis, the study of these gene/protein functions in depth will advance the understanding of their roles in lung cancer cell-derived exosome-mediated signal transduction.

A well-known protein classification system known as PANTHER organized protein classes on the basis of protein functions and included proteins associated with each class [151,152]. In lung cancer exosome studies, proteins encapsulated in exosomes were categorized into the representative PANTHER classes (Table 2). In addition, ExoCarta, which is an exosome database, listed the top 100 proteins that were frequently identified in exosomes derived from multiple sample sources, including various cell types and body fluids [92]. The commonly used exosome markers were indeed in the top 100 protein list. The verification as to whether exosomal proteins from lung cancer samples were in the list of the top 100 proteins could advance the understanding of similarities (i.e., common proteins) and differences (i.e., specific proteins) between lung cancer cell type-derived exosomes and other cell type-derived exosomes.

Proteins and their functions are briefly summarized according to the following classes: (1) tetraspanin family proteins (e.g., CD9, CD63, and CD81), (2) exosome formation-/secretion-required proteins (e.g., ESCRT-associated proteins, Rab family proteins, and annexins), (3) chaperones (e.g., HSPs), (4) structural proteins (e.g., cytoskeletal proteins), (5) cell–microenvironment interactors (e.g., integrins), (6) enzymes and enzyme modulators (e.g., ligase and G-protein modulator), (7) signaling proteins (e.g., growth factors), and (8) immunoregulatory proteins (e.g., immune checkpoint proteins).

Within the tetraspanin family, CD9, CD63, and CD81 are commonly used as exosome markers. Tetraspanins, which are membrane proteins with four transmembrane domains, are known to possess multiple functional roles in several biological processes, such as cell adhesion, fusion, signaling, and trafficking [153]. Evidence has shown that the number of exosomes released from CD9-null bone marrow dendritic cells (BMDCs) is reduced [154]. In contrast, CD9 overexpression in HEK293 cells (human embryonic kidney 293 cells), HEK293FT cells, Raji cells (human B lymphocytes), and Jurkat cells (human T lymphocytes) were found to significantly increased the production and release of exosomes [155]. In addition, another tetraspanin (CD63) was shown to participate in the sorting of a tracked protein (PMEL luminal domain) into ILVs through an ESCRT-independent mechanism [156]. Moreover, it has been reported that a CD63 knockout in HEK293 cells reduced the secretion of EVs [157]. The above evidence indicates that tetraspanins play important roles in exosome biogenesis via an ESCRT-independent mechanism. In addition to being an exosome marker, CD9 was previously demonstrated to participate in the invasive and metastatic abilities of human breast cancer cells [158,159], whereas CD81 has been reported to modulate immune suppression and promote tumor growth and metastasis [160]. Proteins related to the biogenesis and secretion of exosomes (such as ALIX and TSG101) facilitate exosome formation via the ESCRT mechanism [7]. Among chaperones, HSPs are well known for their function in protecting proteins from misfolding and their induction in response to stimuli and stress. HSPs are commonly found in exosomes released from several types of cells [161]. The roles of free and exosome-borne HSPs in cancer development are becoming increasingly clear [162]. Furthermore, Hsp90α, which is carried by cancer cell-derived exosomes, can reportedly increase cancer cell migration [163].

RABs, which are in the GTPase family, are known to modulate membrane trafficking, with roles in endocytosis, exocytosis, and exosome secretion [164,165]. Annexins, which are a family of phospholipid-binding proteins, serve as membrane scaffold proteins to stabilize and organize organelles and plasma membranes and participate in membrane vesicle trafficking, thus suggesting their roles in exosome biogenesis [166]. Annexins (A1, A2, A3, A5, A6, and A11) have been identified in lung cancer exosomes [105,120,132,136]. Annexin A2 in exosomes has been demonstrated to promote angiogenesis and the formation of a microenvironment suitable for breast cancer metastasis [167]. Annexin A2 has also been shown to regulate protein oxidation in a study on ANXA2-depleted cancer cells, ANXA2-null mice, and ex vivo human cancer models [168]. The redox regulatory function of annexin A2 has been shown to protect tumor cells against oxidative stress during tumorigenesis [168]. Among structural proteins, cytoskeletal proteins (such as TPM3 [128] and GCC2 [145]) have been detected in lung cancer exosomes. Cytoskeletal proteins are needed for the transport of multivesicular bodies (MVBs) to the plasma membrane, which is an intermediate process in exosome release [169]. In addition to their role in providing a structural framework, cytoskeletal proteins also regulate cell signaling by mediating receptor dynamics, organization, and crosstalk, thus highlighting their versatile nature [170]. In the tumor microenvironment, ECM–receptor interactions are important for cells, and these interactions can be mediated by integrin receptors. Specific subunit patterns of integrins, which are a family of cell surface receptors with heterodimeric subunits, determine the binding and adhesion preference of cells to distinct ECM proteins within their microenvironment [171,172]. Integrin subunits (α3 and β1) have been discovered in lung cancer exosomes [133]. Integrins are recognized as one of the molecules in exosomes that can regulate tumor cell metastasis; specifically, integrin-α6β1 and integrin-α6β4 promote lung metastasis, whereas integrin-αVβ5 supports liver metastasis [173]. In addition, integrin-αVβ6 and integrin-αVβ3 have been shown to be transferred via prostate cancer cell-derived exosomes to neighboring cells to promote cell migration [174,175].

Several types of enzymes (e.g., HUWE1 [128]) and enzyme modulators (e.g., IQGAP [122]) have been identified in lung cancer exosomes, thus implying that these proteins may be transferred from exosomes to target cells to perform their functions. HUWE1 contributes to lung tumorigenesis, and the mRNA expression of HUWE1 could be used to assess the prognosis of lung cancer patients [176]. IQGAP1 overexpression or mislocalization have been found to be associated with cancer metastasis [177]. As a G-protein modulator, IQGAP1 can modulate the activities of several types of small GTPases, thus leading to cytoskeletal rearrangement, cell proliferation, and migration. Intercellular signaling molecules can trigger signaling pathways of their targeting cells. The signal of AREG, which is an EGFR ligand, has been observed to stimulate cancer cell invasion [178] and activate the EGFR pathway to induce osteoclastogenesis [113,179]. Additionally, mast cell-derived exosomes can transmit TGFβ-1 signals to enhance the migration ability of MSCs [180]. TGF-β has been shown to mediate the EMT in lung cancer cells [181]. Moreover, high TGF-β expression may be an indicator of poor prognosis for lung cancer treatment [182]. For immunoregulation, PD-L1 and Tim-3 are immune checkpoint proteins and can contribute to the regulation of the immune response [183,184]. The development of immune checkpoint inhibitors to block the PD-1/PD-L1 pathway provides a novel strategy for cancer immunotherapy [183,185]. The blockage of Tim-3 and the TIM3/Gal9 pathway could also enhance antitumor immunity for cancer treatment [184,186]. Moreover, Tim-3 and Gal9 were found to be potential biomarkers for the prognosis of various cancers.

Although some proteins were not assigned to suitable protein classes according to the PANTHER classification system, they still have important molecular functions involved in several biological processes. For example, GRB2 is a pivotal protein in signal transduction [187]. Upon the stimulation of external signals (such as EGF-EGFR signaling), the Grb2–Sos complex can be recruited to activate Ras, and active Ras triggers downstream Ras-Raf-MEK-ERK cascades [188,189]. The Ras-Raf-MEK-ERK signaling cascade is an important pathway that regulates cell proliferation and differentiation and contributes to the development of cancers [188,189].

## 7. Lipid Composition and Function of Lung Cancer Exosomes

Exosomes are released by the fusion of MVBs with the cell membrane, thus rendering exosome lipid-enriched membrane structures. Exosome release relies on not only proteins but also lipids that participate in exosome biogenesis and secretion [190]. Although the roles played by lipids have been less frequently reported than those of proteins, the lipid characterization of exosomes can clarify the involvement of lipids in exosome release and uptake.

The lipid bilayer membrane of exosomes contains various lipids to maintain exosome structure and to protect the protein and nucleic acid contents [77]. Exosomal lipid composition was detected in various cell types, including prostate (PC-3), breast (MDA-MB-4175), and pancreatic (AsPC-1) cancer cells; hepatocellular carcinoma (HepG2/C3a and Huh7); glioblastoma (U87); melanoma (B16-F10); MSCs; B-lymphocytes; oligodendroglial precursor cells (Oli-neu); DCs; mast cells (RBL-2H3); adipocytes (3T3-L1); reticulocytes; and platelets [191,192,193]. Cholesterol, sphingomyelin (SM), phosphatidylcholine (PC), PS, phosphatidylethanolamine (PE), phosphatidylinositol (PI), and ceramide are major lipid classes commonly found in exosomes [96,191,192]. Some exosomal lipids have been proposed to be diagnostic biomarkers for prostate, lung, pancreatic, and colorectal cancer, as well as for diabetic nephropathy, multiple sclerosis, and hereditary α-tryptasemia [193].

Lipidomic profiles can reveal the lipid landscape of exosomes. For instance, Llorente et al. quantified 277 lipid species in the membranes of PC-3 cells (human prostate cancer cells) and in the exosomes they release, and they found that glycosphingolipids, SM, cholesterol, and PS were highly enriched in PC-3 cell-derived exosomes, which demonstrated a particular pattern of lipid sorting into these exosomes [194]. Notably, the lipids in the two leaflets of the exosomal bilayer membrane are asymmetrically distributed [191,194], which may enable exosomes to present different outward and inward signals.

In a lung cancer study, Fan et al. explored the lipid profiles of blood plasma exosomes and distinguished 39 normal donors and 91 NSCLC patients (including 44 patients in early stage cancer and 47 patients in late stage cancer) [195]. Lipid features were obtained from MS data of exosome samples. By using the random forest (RF) and least absolute shrinkage and selection operator (LASSO) statistical methods, the authors selected 16 and 7 important features, respectively, out of a total of 430 lipid features to build classification models. Lipid species assigned from the 16 lipid features of the RF model were PC(18:1_18:2), PC(18:0_18:2), PC(16:0_22:6), PC(16:0_18:2), SM(d18:1_16:0), PC(18:0_20:3), PC(16:0_20:4), PC(16:0_22:5), CE(20:4), TAG(52:5), SM(d18:1_24:1), PC(18:0_18:1), PC(16:0_16:0), TAG(54:6), LysoPC(16:0), and LysoPC–pmg(12:0). The RF-based (16-feature) model showed a better performance for the discrimination between normal and late subjects than that between normal and early subjects, whereas the classification between early and late subjects had the lowest performance. In contrast, the LASSO-based (seven-feature) model exhibited classification performance in the order of normal versus early, normal versus late, and then early versus late. This study showed that the exosomal lipid-based classification models were mainly composed of lipid classes of PC, SM, cholesterol esters (CE), triacylglyceride (TAG), and lysophosphatidylcholine (LysoPC) (Table 3), thus implying that these lipid classes were found and detected in exosomes from lung cancer samples and that the lipid features could be lipid biomarkers for the discrimination between lung cancer patients and healthy individuals.

The membrane component SM helps to maintain membrane integrity and stability and is enriched in lipid rafts that help to regulate cellular signaling [196]. Dynamic SM content in lipid rafts has been shown to modulate inflammatory signaling, cholesterol homeostasis, and insulin sensitivity [196]. Lipid raft domains have been found in exosomal membranes, and these lipid microdomains were shown to facilitate molecule sorting during exosome formation [197]. In addition, sphingomyelinase (SMase) catalyzes the conversion of SM to ceramide, which can serve as a second messenger to mediate cell growth, differentiation, and survival [196]. Previously, the inhibition of neutral SMase in mouse oligodendroglial cells (Oli-neu cells) was shown to inhibit the production of ceramide, which considerably reduced exosome release and the amount of a tracked protein (proteolipid protein, or PLP) within the endosomal lumen, thus implying that ceramide participates in the inward budding of endosomes that form ILVs and then MVEs [198], which is an essential process for sorting cellular cargo into exosomes. Moreover, SM in extracellular membrane vesicles derived from tumor cells has been shown to promote endothelial cell migration, tube formation, neovascularization, and angiogenesis during tumor growth and metastasis [199].

## 8. The Importance of CSCs and CSC-Derived Exosomes

Cancer tumors grow in a complicated tumor microenvironment with multiple types of cells, such as cancer cells, CSCs, cancer-associated fibroblasts (CAFs), immune cells, and other normal cells [10]. Conventional cancer treatment involves a tumor cell-targeting approach by which differentiated tumor cells are killed; however, the remaining CSCs survive [200]. Resistant CSCs may initiate tumor regrowth and drive cancer relapse [200]. CSC-targeting therapy is an innovative cancer treatment that directly kills CSCs to ultimately eradicate tumors on the basis of the premise that tumor cells have limited proliferation and self-renewal abilities [200]. A combinatorial treatment with anticancer cell and anti-CSC drugs may improve cancer treatment.

As CSCs play important roles in tumor recurrence and drug resistance, they may transmit crucial signaling to other cells via exosomes. EV-mediated crosstalk between CSCs and molecules in the tumor microenvironment can influence the fate of normal and tumor cells, thereby regulating solid tumor progression [201]. However, only a few studies have investigated exosomes derived from lung CSCs. A comprehensive molecular characterization of lung CSC-derived exosomes and the mechanisms involved in tumor progression has remained elusive [202]. The greatest challenge to characterize exosomes is obtaining sufficient amounts of CSCs and exosomes to study. To study CSCs and CSC-derived exosomes, CSCs should be first isolated from the heterogeneous microenvironment to reduce signal noise from other types of cells (i.e., exosome signals from non-CSC cells). CSCs can be isolated and characterized by cell sorting by using surface markers and several functional assays, including in vitro spheroid and colony formation assays, side population assays, aldehyde dehydrogenase (ALDH) activity assays, and in vivo tumorigenicity assays [203]. Various CSC markers are found in different types of solid tumors [204,205]. For lung cancer, CD133, CD44, CD90, CD166 (ALCAM), CD177, CD87, ALDH1A1, ABCG2, EpCAM, and CXCR4 have been reported to be lung CSC markers [204,205,206]. Through one or more surface markers and cell sorting, lung CSCs can be isolated and enriched from the mixed cell types of tumors. Further characterizations of tumorigenicity, self-renewal, and differentiation abilities are required to verify their CSC properties. For example, the isolation and characterization of CSCs from the lung cancer cell line A549 have been demonstrated, including cell culture, cell sorting, differentiation assay, clonogenic assay, spheroid formation assay, and tumorigenicity assay [207]. One miRNA study in lung CSC-derived exosomes demonstrated that A549 cell-derived tumor spheres have multiple stemness characteristics and could be used as lung CSCs [208]. This study showed that lung CSC-derived exosomes could enhance lung cancer cell migration and invasion, and exosomal miR-210-3p could regulate the metastatic ability of lung cancer cells.

Several exosomal miRNAs from various CSCs (including lung, breast, colorectal, gastric, pancreatic, prostate, renal, glioblastoma, oral squamous cell carcinoma, and papillary carcinoma) have been found [209]. A few miRNAs of lung CSC-derived exosomes have been investigated [208,210]. In comparison with miRNAs, proteins and lipids in lung CSC-derived exosomes are less discussed. Therefore, more studies of lung CSC-derived exosomes are in demand. It would be useful to identify exosome signals solely from CSCs and to characterize the difference between CSC-derived exosomes and non-CSC-derived exosomes. This would facilitate the development of CSC-targeting therapy for lung cancer, as well as other cancers.

## 9. Conclusions

Exosomes are cell–cell communicators within the tumor microenvironment. The molecular cargo carried by exosomes represents the content of the cells of origin and shows functional potential for cancer cell-mediated signaling. The identification of the molecular components of exosomes can help to pinpoint a cell–cell communication mechanism and may eventually lead to the identification of potential diagnostic and prognostic biomarkers for cancer detection and disease staging.

This review presents current potential exosomal proteins and lipids for the development of lung cancer detection, diagnosis, treatment, prognosis, and follow-up monitoring. We organized lung cancer-derived exosomal proteins and lipids from the available literature, regardless of sample source. Exosomes were isolated and purified from various lung cancer sample sources, including lung cancer cell lines and liquid biopsies, such as blood (plasma and serum), urine, and saliva.

Evidence has shown that exosomal protein and lipid cargos contain components necessary for exosome formation, structural components related to the cytoskeleton, and signaling molecules for intercellular communication and regulation. Proteins participate in several biological processes through different pathways and networks to regulate other molecules and to exert their own molecular functions. Enzymes and enzyme modulators help substrates to transform to active or inactive forms to control molecular transformation and metabolism during bioactive signaling. Structural proteins maintain cell structure and are packaged into exosomal cargo; they may also play versatile functional roles in mediating receptor dynamics and organization. Moreover, tetraspanin family proteins play multiple roles in the regulation of several biological processes, thus leading to changes in tumor cell growth, invasiveness, and metastatic capacity. Exosome formation-/secretion-required proteins participate in the process of exosome biogenesis and release and are involved in exosome internalization into target cells. Additionally, cell microenvironment interactors coordinate cell interactions and communication with the external environment to form a suitable niche for cell adhesion and migration. Signaling proteins transmit signals to target cells to modulate recipient cell molecular networks and can consequently affect their cellular behavior. Furthermore, chaperones protect proteins from misfolding and are important to cancer development and progression. In summary, several pathways are involved in transmitting signals from donor cells to recipient cells via exosomes, including the endocytic pathway; endosomal sorting- and transportation-related interaction pathways for cargo packaging and exosome release; exosome- and microenvironment-related pathways that promote cell adhesion and targeting; and pathways involved in exosome uptake that enable signal transduction and the promotion of enzymatic activity within target cells. In contrast, lipids not only play structural roles in maintaining exosomal membrane structure but also serve functional roles in exosome biogenesis and uptake, as well as signal modulation. The lipid bilayer membrane structure of exosomes functions as a protector of exosomal protein and nucleic acid contents.

Although exosomes can be released from multiple types of cells within the tumor microenvironment, it is challenging to discriminate the original cell source of exosomes. The CSC subpopulation contributes to tumor progression and is an important source of exosomes. Nevertheless, to the best of our knowledge, after a comprehensive literature review, few or no studies have discussed the proteins and lipids of lung CSC-derived exosomes. The exploration of proteins and lipids, as well as miRNAs, in lung CSC-derived exosomes for lung cancer therapy is still an unmet need. Accordingly, we highlighted the importance of CSCs and CSC-derived exosomes in cancer therapy and listed feasible strategies to obtain CSCs for exosome study.

Cancer cells and CSCs transmit tumor cell signals by packaging essential molecular components into exosomes that are carried to target cells and the tumor microenvironment, wherein the delivered exosome components can change cellular behavior and form an educated niche suitable for tumor growth and progression. Therefore, the characterization of the molecular components and functions of cancer cell- and CSC-derived exosomes helps to understand exosome-associated molecular mechanisms and to discover potential drug(s) for blocking cancer cell- and CSC-derived molecular signals.

In summary, we elaborated on the comprehensive protein and lipid composition and function of lung cancer-derived exosomes. Exosomal molecules could be potential diagnostic and prognostic biomarkers and therapeutic targets for drug development. This review provides a molecular foundation for future studies and applications in exosome-based cancer diagnosis, prognosis, and treatment, especially for lung cancer.

## Figures and Tables

**Table 1:** Protein composition and application of lung cancer exosomes.

Protein		Source		Application ^*b*^	Year [Ref]
Marker	Lung Cancer ^*a*^	Cell Line	Biopsy		
CD63	CD54, CD86, MHC-I, MHC-II, HSP60, HSC70, HSP70, HSP90 (ANXA2, CD18, CD71, CD80, CD107a)	3LL (mouse)	-	Treatment	2011 [105]
	CCL2, CCL3, CCL4, CCL5, CCL20 ^*c*^				
tsg101 ^*d*^	Hsp72 (Hsc73)	H23 (human)	-	Treatment	2010 [106]
CD63	HSP70	A549 (human)	-	Treatment	2016 [107]
CD9 CD63 TSG101	HSP70	-	Plasma (AC) Plasma (SCC)	Diagnosis Prognosis	2020 [108]
FLOT1	EGFR	A549 (human)	-	Treatment	2009 [109]
EEA1	EGFR	-	Tissue (NSCLC)	Treatment	2013 [110]
CD81	EGFR	HARA (human) HARA-B (human) A549 (human) RERF-LC-MS (human) LU65 (human)	Plasma (xenograft mouse) Plasma (human)	Diagnosis	2013 [111]
CD9 CD63	EGFR	A549 (human)	Blood (NSCLC)	Diagnosis Treatment	2019 [112]
CD63 ALIX TSG101	AREG	CRL-2868 (human) A549 (human)	Plasma (NSCLC)	Treatment	2017 [113]
CD9 CD81	CD91 (CD317, BAIP2, CPNE1, FIBA, CO5, MASP1, APOB, CO8A, PLMN, PGBM, PERM, FCGBP, ITA2B, KV313, IC1, A2MG, HABP2)	-	Serum (AC) Serum (SCC)	Diagnosis	2014 [114]
CD9 CD63 CD81 TSG101 Hsp90	30-marker panel: 11 markers, 3 markers (normalized value), 16 marker relations	-	Plasma (AC IIIa-IV)	Diagnosis	2015 [115]
	18 proteins involved: CD81, TAG72, CD63, TSG101, CD9, CD163, MUC1, N-cadherin, EGFRvIII, c-MET, Flotillin-1, HB-EGF, EGFR, TNF RI, NY-ESO-1, CD146, CD142, PLAP				
CD9 CD63 CD81	Single marker: (Cancer vs. noncancer patients) AC, SCC, SCLC: CD151 AC: CD171 AC, SCC: TSPAN8	-	Plasma (AC) Plasma (SCC) Plasma (SCLC)	Diagnosis	2016 [116]
	Multiple markers: (Cancer vs. noncancer patients) AC, SCC, SCLC: CD151, TSPAN8, NY-ESO-1, HER2, CD171, EGFRvIII, SFTPD, Flotillin-1, CD142, Mucin16 AC: CD151, TSPAN8, CD171, SFTPD, CD82, PLAP, NY-ESO-1, HER2, Flotillin-1, Mucin16				
CD9 CD63 CD81 CD82 CD37 Alix TSG101 Hsp90	Presence or not: CD171 (Flotillin-1, HER3, GRP78)	-	Plasma (NSCLC)	Diagnosis Prognosis	2016 [117]
	Expression level: NY-ESO-1, EGFR, PLAP (EpCam, CAIX, CD13, CD276)				
	Measurable level: NY-ESO-1, EGFR, PLAP, EpCam, Alix (HER3, CAIX, CD13, CD276)				
Ezrin β-Actin CD63	912 proteins (e.g., EGFR, KRAS, CEACAM6, BSG, CLDN1, CLDN3, RAB family proteins)	-	Pleural effusion (NSCLC)	Diagnosis Treatment	2013 [118]
CD63 ALIX	A549 & HCC827: EGFR, SRC, GRB2, JAK1 (DSG2, CD151, CNTN1, EDIL3, ITGB6, ITGB1, BSG, SLC3A2, LAMP2, CEACAM6, PAPPA, CTSA, GGT3P)	A549 (human) HCC827 (human)	-	Diagnosis	2016 [119]
	A549: (MFGE8, HSPG2, GPRC5A)				
	HCC827: RALA, CTNNB1 (SDC1, EPCAM, MPZL1, MPZL2, TACSTD2, GNB1, TOM1L1)				
-	ANXA1, ANXA2, ANXA3, ANXA5, ANXA6, ANXA11, NPRL2, CEACAM1, MUC1, PROM1, HIST1H4A, TNFAIP3	-	Saliva (cancer)	Diagnosis	2016 [120]
CD63 TSG101	A1AG1, AQP5, MUC5B	-	Saliva (cancer) Serum (cancer)	Diagnosis	2017 [121]
CD63 CD81 TSG101	MUC5B, IQGAP (ENO1, SPARCL1)	-	Saliva (cancer)	Diagnosis	2018 [122]
CD9 CD63 CD81 TSG101	HGF	95C (LCC) 95D (LCC)	Plasma (xenograft mouse) Plasma (cancer)	Prognosis Treatment	2019 [123]
CD9 CD81 HSP70	ANGPTL4 (ACLY, LRP1, PSMD14, TKT, TTN, VCAN)	A549 (NSCLC)	-	Diagnosis Treatment	2020 [124]
CD9 CD81 TSG101	HTATIP2 RHOV	A549 and others (human)	Tissue (AC) Plasma (AC)	Diagnosis	2020 [125]
-	TGF-β, IL-10	NCI-H1688 (SCLC) NCI-H2228 (NSCLC)	-	Treatment	2016 [126]
CD9 CD63 HSP70	VIM	PC14 (nonmetastatic) PC14HM (highly metastatic)	Serum (early stage) Serum (late stage)	Treatment	2016 [127]
CD9 TSG101	SRGN, TPM3, THBS1, HUWE1 (CCDC18, ALDH1L1, HIST1H4A, NCCRP1, MED14, BHMT, GLUD1, PPIA, EXOC8)	H23 (human) H647 (human) H1573 (human) HCC4019 (human)	Plasma (AC)	Diagnosis	2017 [128]
CD63	Tim-3, Galectin-9	-	Plasma (NSCLC)	Diagnosis Prognosis	2018 [129]
HSP70	LBP	-	Serum (nonmetastatic NSCLC) Serum (metastatic NSCLC)	Diagnosis	2018 [130]
CD9 CD63 CD81 HSP70	AHSG, ECM1	-	Serum (NSCLC)	Diagnosis	2019 [131]
CD63	MUC1 (THBS1, ANXA6, HIST1H4A, COL18A1, MDK, CD151, SRGN, ENO1, TUBA4A, SLC3A2, GPI, MIF, TALDO1, SLC7A5, ICAM1, HSP90AA1, G6PD, LRP1)	NCI-H838 (NSCLC)	Plasma (NSCLC)	Diagnosis	2019 [132]
CD63	ITGA3, ITGB1	A549 (AC) H1975 (AC) H3255 (AC) H1650 (AC)	Pericardial effusion (NSCLC)	Diagnosis	2019 [133]
-	LRG1	-	Urine (NSCLC)	Diagnosis	2011 [134]
CD63 TSG101	LRG1	SPCA1 (NSCLC) A549 (NSCLC) PC9 (NSCLC) H1299 (NSCLC) H358 (NSCLC)	Plasma (NSCLC)	Diagnosis Prognosis Treatment	2019 [135]
CD63 ANXA5	HLA-class I, BAGE, PD-L1, ANXA2	-	BAL (NSCLC)	Diagnosis	2019 [136]
CD63 HRS Alix TSG101	PD-L1	H1299 (human) H358 (human) H1264 (human)	-	Prognosis Treatment	2018 [137]
CD9 HSP70 HSP90	PD-L1	A549 (NSCLC) H460 (NSCLC) H1975 (NSCLC)	Plasma (NSCLC)	Diagnosis	2019 [138]
CD9 CD63	PD-L1	-	Plasma (advanced NSCLC)	Prognosis	2021 [139]
CD63 TSG101	PD-L1	-	Serum (NSCLC)	Diagnosis Prognosis	2019 [140]
-	PD-L1	-	Serum (NSCLC)	Prognosis	2021 [141]
Epcam	PD-L1	A549 (human) SK-MES1 (human)	Plasma (cancer)	Diagnosis Prognosis	2021 [142]
EpCAM	PD-L1	A549 (human) H1975 (human)	Plasma (NSCLC)	Diagnosis	2021 [143]
CD9 CD63 CD81 Alix TSG101	CD5L (CLEC3B, SERFINF1, ITIH4, SAA4, SERFINC1, C20ORF3)	-	Serum (AC) Serum (SCC) Serum (SCLC)	Diagnosis	2021 [144]
CD9 CD63 CD81	GCC2 (TUBA1C, GAPDH, KRT25, POTEKP)	A549 (NSCLC) H1299 (NSCLC) PC9 (NSCLC) H1650 (NSCLC) H522 (NSCLC)	Plasma (AC)	Diagnosis	2021 [145]
CD9 CD63 TSG101	eIF4E ^*e*^	-	Serum (NSCLC)	Prognosis	2020 [146]

*^a^* Proteins listed in parentheses were identified in exosomes from lung cancer samples, although they were not the major focuses or biomarkers in the original papers. However, some of the proteins were still mentioned to be valuable for further investigation. *^b^* Please refer to the section “Protein composition of lung cancer exosomes” and original papers for marker usage. *^c^* Chemokines were enriched in heat-stressed tumor cell-derived exosomes. *^d^* tsg101 expression was confirmed in exosomes from one mouse cell line (EL4 thymoma) that was discussed in the author’s original paper; no evidence was shown in exosomes from the H23 human cell line. *^e^* RNA expression was discussed, although protein expression in exosomes was confirmed. Abbreviations: NSCLC: non-small-cell lung cancer; AC: adenocarcinoma; SCC: squamous cell carcinoma; LCC: large cell carcinoma; SCLC: small cell lung cancer; BAL: bronchoalveolar lavage; A1AG1: alpha-1-acid glycoprotein 1; AHSG: alpha-2-HS-glycoprotein; ANGPTL4: angiopoietin like 4; ANXA1: annexin A1, annexin I; ANXA2: annexin A2, annexin II; ANXA3: annexin A3, annexin III; ANXA5: annexin A5, annexin V; ANXA6: annexin A6, annexin VI; ANXA11: annexin A11, annexin XI; AQP5: aquaporin 5; AREG: amphiregulin; BAGE: B melanoma antigen; CCL: C-C motif chemokine ligand; CEACAM1: carcinoembryonic antigen-related cell adhesion molecule 1; CTNNB1: catenin beta 1; ECM1: extracellular matrix protein 1; EEA1: early endosome antigen 1; EGFR: epidermal growth factor receptor; eIF4E: eukaryotic translation initiation factor 4E; EpCAM: epithelial cell adhesion molecule; FLOT1: flotillin 1; GCC2: GRIP and coiled-coil domain-containing protein 2; GRB2: growth factor receptor bound protein 2; HGF: hepatocyte growth factor; HIST1H4A: histone H4; HLA: human leukocyte antigen; HRS: hepatocyte growth factor-regulated tyrosine kinase substrate; HSC: heat shock cognate protein; HSP: heat shock protein; HTATIP2: HIV-1 Tat interactive protein 2; HUWE1: HECT, UBA, and WWE domain-containing E3 ubiquitin protein ligase 1; IL-10: interleukin 10; ITGA3: integrin subunit alpha 3; ITGB1: integrin subunit beta 1; JAK1: Janus kinase 1; LBP: lipopolysaccharide-binding protein; LRG1: leucine-rich-alpha2-glycoprotein 1; MHC: major histocompatibility complex; MUC1: mucin 1; MUC5B: mucin 5B; NPRL2: nitrogen permease regulator 2-like protein; PD-L1: programmed death-ligand 1; PLAP: placental alkaline phosphatase; PROM1: prominin-1; RALA: RAS like proto-oncogene A; RHOV: rho-related GTP-binding protein RhoV; SRC: SRC proto-oncogene, non-receptor tyrosine kinase; SRGN: serglycin; TGF-β: transforming growth factor beta 1; THBS1: thrombospondin 1; Tim-3: T cell immunoglobulin and mucin domain-containing protein 3; TNFAIP3: tumor necrosis factor alpha-induced protein 3; TPM3: tropomyosin alpha-3 chain; TSG101: tumor susceptibility gene 101; TSPAN8: tetraspanin 8; VIM: vimentin. Alias: LCC/LCLC (large cell lung carcinoma); CRL-2868/HCC827; A1AG1/ORM1 (orosomucoid 1); ALIX/PDCD6IP (programmed cell death 6 interacting protein); CD54/ICAM1 (intercellular adhesion molecule 1); CD91/LRP1 (LDL receptor related protein 1); CD151/TSPAN24 (tetraspanin-24); CD171/L1CAM (L1 cell adhesion molecule); eIF4E/eIF-4E/EIF4E; Galectin-9/LGALS9; HIST1H4A/H4C1 (H4 clustered histone 1); HRS/HGS; HSC70/HSPA8; HSP90/HSP90AA1; IL-10/IL10; IQGAP/IQGAP1 (IQ motif containing GTPase-activating protein 1); NY-ESO-1/ESO1/CTAG1B (cancer/testis antigen 1B); PD-L1/CD274; PLAP/ALPP (alkaline phosphatase, placental); TGF-β/TGFB1; Tim-3/TIM3/HAVCR2 (hepatitis A virus cellular receptor 2).

**Table 2:** Protein, protein class, and subclass of lung cancer exosomes.

Protein Class ^*a*^	Protein Subclass ^*a*^	Protein	Top100 ^*c*^
Calcium-binding protein	-	ANXA1	Yes
		ANXA2	Yes
		ANXA3	No
		ANXA5	Yes
		ANXA6	Yes
		ANXA11	Yes
Cell adhesion molecule	integrin	ITGA3	No
		ITGB1	Yes
	-	MUC1	No
Chaperone	Hsp90 family chaperone	HSP90/HSP90AA1	Yes
Chromatin/chromatin-binding, or -regulatory protein	histone	HIST1H4A/H4C1	Yes
Cytoskeletal protein	microtubule or microtubule-binding cytoskeletal protein	GCC2	No
	actin binding motor protein (actin or actin-binding cytoskeletal protein)	TPM3	No
Defense/immunity protein	immunoglobulin receptor superfamily	CD86	No
		PD-L1/CD274	No
		Tim-3/TIM3	No
	major histocompatibility complex protein	MHC-I	No
		MHC-II	No
Extracellular matrix protein	-	Galectin-9	No
		MUC5B	No
Intercellular signal molecule	-	ANGPTL4	No
	growth factor	AREG	No
		TGF-β/TGFB1	No
Membrane traffic protein	-	ALIX/PDCD6IP	Yes
Metabolite interconversion enzyme	oxidoreductase	HTATIP2	No
	phosphatase (hydrolase)	PLAP/ALPP	No
Protein modifying enzyme	serine protease (protease)	HGF	No
	cysteine protease (protease)	TNFAIP3	No
	ubiquitin-protein ligase	HUWE1	No
		TSG101	Yes
Protein-binding activity modulator	protease inhibitor	AHSG	No
	GTPase-activating protein (G-protein modulator)	IQGAP/IQGAP1	No ^*d*^
		NPRL2	No
	small GTPase (G-protein)	RALA	No ^*d*^
		RHOV	No
Transfer/carrier protein	apolipoprotein	CD91/LRP1	No
Transmembrane signal receptor	-	EGFR	No
Transporter	-	AQP5	No
NA ^*b*^	NA ^*b*^	CD9, CD63, CD81, FLOT1, HSC70/HSPA8, HSP70, Hsp72, THBS1	Yes
		A1AG1/ORM1, BAGE, CD5L, CD54/ICAM1, CD151, CD171/L1CAM, CEACAM1, CTNNB1, ECM1, EpCam, GRB2, HRS/HGS, HSP60, IL-10/IL10, JAK1, LBP, LRG1, NY-ESO-1/ESO1/CTAG1B, PROM1, SRC, SRGN, TSPAN8, VIM	No

*^a^* Protein class and subclass were based on PANTHER classification system (http://www.pantherdb.org/) (accessed on 27 December 2021). *^b^* NA: Protein class and subclass were not available for the list of proteins. *^c^* Yes/No: Protein found in top100 proteins of ExoCarta (http://www.exocarta.org/) (accessed on 27 December 2021) or not. *^d^* Protein not found in top100 proteins of ExoCarta but found in top100 EV proteins of Vesiclepedia (http://www.microvesicles.org/) (accessed on 27 December 2021).

**Table 3:** Lipid composition and application of lung cancer exosomes.

Lipid		Source		Application ^*b*^	Year [Ref]
Marker	Lung Cancer	Cell Line	Biopsy		
-	16 lipid features ^*b*^: PC(18:1_18:2), PC(18:0_18:2), PC(16:0_22:6), PC(16:0_18:2), SM(d18:1_16:0), PC(18:0_20:3), PC(16:0_20:4), PC(16:0_22:5), CE(20:4), TAG(52:5), SM(d18:1_24:1), PC(18:0_18:1), PC(16:0_16:0), TAG(54:6), LysoPC(16:0), LysoPC–pmg(12:0)	-	Plasma (NSCLC I, II) Plasma (NSCLC III, IV)	Diagnosis	2018 [195]

*^a^* Classification models were built on the basis of lipid features (monoisotopic accurate *m*/*z* values). The assigned lipid names were listed. *^b^* Please refer to the section “Lipid composition and function of lung cancer exosomes” and original papers for marker usage. Abbreviations: NSCLC: non-small-cell lung cancer; CE: cholesterol ester; LysoPC: lysophosphatidylcholine; LysoPC–pmg: lysophosphatidylcholine-plasmalogen; PC: phosphatidylcholine; SM: sphingomyelin; TAG: triacylglyceride.

## Data Availability

No new data were created or analyzed in this study. Data sharing is not applicable to this article.

## Abbreviations

AC: adenocarcinoma; AFM: atomic force microscopy; ALDH: aldehyde dehydrogenase; AUC: area under the curve; BAL: bronchoalveolar lavage; BMDC: bone marrow dendritic cell; CAF: cancer-associated fibroblast; CE: cholesterol ester; CEA: carcinoembryonic antigen; CSC: cancer stem cell; CTC: circulating tumor cell; DC: dendritic cell; DLS: dynamic light scattering; ECM: extracellular matrix; ELISA: enzyme-linked immunosorbent assay; EM: electron microscopy; EMT: epithelial–mesenchymal transition; ESCRT: endosomal sorting complex required for transport; EV: extracellular vesicle; EVP: extracellular vesicle and particle; GAG: glycosaminoglycan; HBEC: human bronchial epithelial cell; HPAEpiC: human pulmonary alveolar epithelial cell; HSP: heat shock protein; HUVEC: human umbilical vein endothelial cell; ICI: immune checkpoint inhibitor; ILV: intraluminal vesicle; LASSO: least absolute shrinkage and selection operator; LCC: large cell carcinoma; lncRNA: long noncoding RNA; LysoPC: lysophosphatidylcholine; MDSC: myeloid-derived suppressor cell; miRNA: microRNA; MS: mass spectrometry; MSC: mesenchymal stem cell; MV: microvesicle; MVB: multivesicular body; MVE: multivesicular endosome; NF-κB: nuclear factor-kappa B; NSCLC: non-small-cell lung cancer; NTA: nanoparticle tracking analysis; OS: overall survival; PC: phosphatidylcholine; PE: phosphatidylethanolamine; PFS: progression-free survival; PI: phosphatidylinositol; PLP: proteolipid protein; PS: phosphatidylserine; RF: random forest; ROC: receiver operating characteristic; SCC: squamous cell carcinoma; SCLC: small cell lung cancer; Simoa: single molecule array; SM: sphingomyelin; SMase: sphingomyelinase; TAG: triacylglyceride; TNBC: triple-negative breast cancer; VEGF: vascular endothelial growth factor; VEGFR-2: vascular endothelial growth factor receptor-2; WHO: World Health Organization.
